# Methylation analysis of the phosphates and tensin homologue on chromosome 10 gene (*PTEN*) in multiple myeloma

**DOI:** 10.1186/1868-7083-6-16

**Published:** 2014-08-20

**Authors:** Giovanna Piras, Maria Monne, Angelo D Palmas, Anna Calvisi, Rosanna Asproni, Francesco Vacca, Laura Pilo, Attilio Gabbas, Giancarlo Latte

**Affiliations:** 1 UOC Ematologia, Ospedale “San Francesco”, ASL Nuoro. Via Mannironi 1, Nuoro, 08100, Italy

**Keywords:** Promoter hypermethylation, multiple myeloma, Akt pathway, *PTEN*

## Abstract

**Background:**

Aberrant DNA methylation of promoter region CpG islands is an alternative mechanism that leads to genetic defects in the inactivation of tumor suppressor genes during myelomagenesis. The aim of this study was to examine the promoter methylation status of the phosphates and tensin homologue on chromosome 10 (*PTEN*) gene in a cohort of multiple myeloma patients.

**Findings:**

*The PTEN* gene was hypermethylated in 7 out of 58 (12%) primary myeloma samples. The correlation between functional inactivation and *PTEN* mRNA levels was not statistically significant. The multiple myeloma subgroup with an aberrant *PTEN* status had a prevalence of the component IgG, Salmon Durie stage I, lower lactate dehydrogenase levels, intermediate-standard cytogenetic risk and longer overall survival with the respect to the unmethylated subgroup.

**Conclusions:**

This is the first report demonstrating the presence of *PTEN* promoter hypermethylation in multiple myeloma.

## Findings

### Background

Multiple myeloma (MM) is a B-cell neoplasm characterized by end-organ damage, which may include hypercalcemia, renal dysfunction, anemia and lytic bone lesions. MM comprises various biology entities with a heterogeneous clinical course, ranging from a relatively benign disorder to a highly aggressive disease with rapid progression. The MM pathogenesis is a multistage, complex process where cytogenetic, molecular and epigenetic mechanisms act together. It has been reported that during myelomagenesis, DNA hypomethylation is the predominant early change that is gradually transformed to DNA hypermethylation in relapsed cases and during the progression of the disease [1,2]. Several malignancy-related genes (*VHL, XAF1, IRF8, TP53, CDKN2A-P16, CDKN2B, DAPK, SOCS1, CDH1, PTGS2, CCND2* and *WNT* inhibitor genes) that affect various molecular pathways were shown to be hypermethylated in MM, indicating that they contribute to tumor formation and progression [3-5]. Promoter hypermethylation of *CDKN2A*, which is a marker for overall epigenetic changes, and *TGFBR2* have been shown to correlate with poor prognosis in MM patients, although the prognostic value of *CDKN2A* hypermethylation remains debatable [6]. Hypermethylation of *GPX3, RBP1, SPARC* and *TGFB1* genes was also demonstrated to be associated with shorter overall survival.

The phosphates and tensin homologue on chromosome 10 gene (*PTEN*) is a tumor-suppressor gene known to be mutated in multiple cancer [7,8]. *PTEN* regulates the cell cycle progression, apoptosis, metastasis and invasion of the tumor cells by negatively controlling the PI3P/Akt pathway. It can be inactivated by mutation, loss of heterozygosity, promoter hypermethylation, abnormal micro-RNA expression and post-translational phosphorylation [9]. *PTEN* alterations are detected, though sparsely, in MM patients with advanced disease [10]. Abnormal expression of *PTEN* in myeloma cell lines and MM patients has been reported and may be associated with disease progression and extramedullary infiltration [11,12]. So far, *PTEN* deregulation has not been proven to be due to epigenetic silencing. This study analyzed the occurrence and the possible impact of *PTEN* epigenetic status in a cohort of multiple myeloma patients.

### Patients and methods

#### Clinical specimens

Bone marrow (BM) aspirates and clinical data from 58 patients with MM (30 male, 28 female; median age 67.8 years, range 29 to 86) were obtained during routine clinical assessment at time of first diagnosis when the patients were admitted to our hospital between 2000 and 2008, and for some, as the disease progressed. MM diagnosis was made in accordance with standard criteria, and staging was according to the Salmon Durie Staging System. MM clinical characteristics and laboratory parameters are reported in Table 1. Forty-six out of 58 (79.8%) MM patients had an IgG monoclonal component, and 46.5% of patients were assigned Salmon Durie stage III. Cytogenetic analysis data allowed us to categorize 62.1% of the patients as high cytogenetic risk, defined by the presence of t(4;14), t(14;16) or 17p13 deletion by fluorescent in situ hybridization (FISH) analysis, while the remaining patients were considered at intermediate (presence of 1q gain, complex karyotype, deletion 13) or standard risk (hyperdiploidy, t(11;14)) [13]. A total of 52 bone marrow aspirates from 12 patients with monoclonal gammopathy of undetermined significance (MGUS), 10 with acute myeloid leukemia (AML), 10 with chronic myeloid leukemia (CML), 10 with acute B lymphoblastic leukemia (B-ALL) and 10 with myeloproliferative disorders (MPD) were collected as part of the routine staging procedure and analyzed. Seven bone marrow aspirates obtained from patients with malignant lymphoma without bone marrow infiltration were used as control samples. The ASL Nuoro ethics committee approval and patient informed consent were obtained.

**Table 1:** Association of clinical and laboratory parameters with PTEN methylation status

**Parameter**	**PTEN unmethylated n = 51**	**PTEN methylated n = 7**	***P*** **value**
**Heavy chain**			0.8
IgG	40	6	
IgA	10	1	
IgM	1	0	
**Light chain**			0.04
Kappa	25	7	
Lambda	16	0	
**Stage**			
I	14	4	0.3
II	10	1	
III	25	2	
**Age (mean in years)**	67.6 ± 13.0	69 ± 10.1	0.7
**White blood cells (mean n°x10^3/mcL)**	6.58 ± 2.2	6.0 ± 2.9	0.5
**Hemoglobin (mean in g/dL)**	10.2 ± 2.1	11.2 ± 1.9	0.2
**Platelets (mean n°x10^3/mcL)**	235 ± 95	210 ± 65	0.5
**Lactate dehydrogenase (mean in U/L)**	379 ± 186	200 ± 40	**0.01**
**β2-microglobuline (mg/dL)**	5.3 ± 4.5	5.3 ± 5.4	0.9
**Albumin (mean in g/dL)**	3.6 ± 0.7	3.9 ± 0.8	0.3
**Serum creatinine (mean in mg/dL)**	2.0 ± 2.35	1.0 ± 0.6	0.2
**Calcium (mean in mg/dL)**	6.0 ± 2.5	7.1 ± 2.5	0.3
**Protein (mean in g/L)**	8.5 ± 1.8	8.8 ± 1	0.66
**Plasma cells (mean in %)**	52 ± 31	58 ± 24	0.6
**Cytogenetic risk**			0.68
Standard	31	5	
Intermediate	15	1	
High	5	1	

#### Methylation analysis

High molecular weight DNA was isolated by the salting-out method from bone marrow aspirates and was treated with bisulphite for conversion of unmethylated but not methylated cytosine to uracil using the CpGenome DNA modification kit (Chemicon, Millipore SPA, (Italy). Methylation-specific polymerase chain reaction (MSP) was performed as initially described by Herman *et al.* 1996 [14]. Primers for the methylated and unmethylated *PTEN* gene promoter regions, which amplified a region of 187 bp, were used [15]. In all experiments, DNA from lymphoma specimens without bone marrow infiltration were used as the negative control sample, and methylated control DNA (CpGenome Universal Methylated DNA, Chemicon, Millipore SPA, Italy) was used as the positive control. PCR products were analyzed on 2% agarose gels and visualized under ultraviolet illumination.

#### Expression analysis

In a subset of 31 MM bone marrow specimens, analyzed for PTEN gene promoter hypermethylation, total RNA was isolated using Trizol reagent. The PTEN relative expression level was analyzed in duplicate by quantitative reverse transcriptase PCR using the TAMRA Probe approach on ABI Prism 7500 (PE Applied Biosystems, Foster City, CA). PTEN primers were: forward 5’- AAGACATTATGACACCGCCAAAT-3’ reverse 5’- ATGATTGTCATCTTCACTTAGCCATT-3’. Probe: TET-TGCAGAGTTGCACAATATCCTTTTGAAGACC-TAMRA [16] Quantitative data were normalized to Abelson (*ABL1*) as reference gene, and the 2-∆∆Ct method was used for data analysis. Ten samples from MGUS patients and six peripheral blood samples from controls were also evaluated.

#### Statistical analysis

Differences between groups were analyzed using the chi-squared test and were considered significant for *P* value <0.05. The Kaplan-Meier method was used to estimate the probability of survival as function of time. Survival differences among comparator groups were analyzed by the log-rank test. Statistical analyses were done using SPSS 7.0 statistical software.

## Results

**Figure 1:**
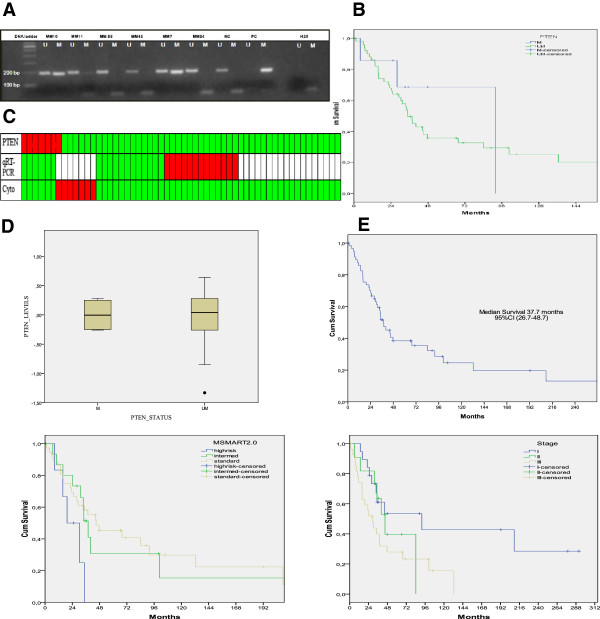
***PTEN*gene hypermethylation analysis in multiple myeloma patients. (A)** Representative methylation specific polymerase chain reaction (MSP) results for PTEN in patient samples. The positive and negative controls showed the expected MSP results with normal DNA showing positive U-MSP but negative M-MSP amplification, and conversely, the methylated control DNA showing negative U-MSP but positive M-MSP amplification. **(B)** Kaplan-Meyer survival function of multiple myeloma (MM) patients with or without PTEN promoter hypermethylation, *P* value = 0.53. **(C)** Heatmap of the PTEN analyses showing methylation status, quantitative reverse transcriptase PCR (qRT-PCR) for PTEN relative gene expression and cytogenetic categories. MSP green, unmethylated; red, methylated; qRT-PCR: green, low expression; red, high expression and white, not determined. Cytogenetics: green, standard risk; red, high risk. **(D)** Box and whisker plot showed the PTEN expression in methylated and unmethylated primary MM patients, *P* value = 0.87. **(E)** Survival curves of 58 patients with multiple myeloma according to SD staging (*P* = 0.053), and cytogenetic categories (*P* = 0.11).

Among the MM samples, the median overall survival time (OS) was 37.7 months (Figure 1E). Moreover, the median OS of patients with SD stage I, II and III were 91.8, 44.9, and 29.0 months, respectively (*P* = 0.039), thereby suggesting that our MM patient group was an unbiased myeloma cohort (Figure 1E). Stratification of the MM patients according to cytogenetic risk categories showed a median OS of 44.9 months for the standard risk, 37.9 months for the intermediate risk and 19.3 months for the high risk group (*P* = 0.121).

MSP was used to analyze the methylation status of PTEN in 58 MM, 52 patients with other hematological malignancies and 7 ‘*normal*’ BM samples obtained from non-infiltrated lymphomas. Aberrant promoter methylation was detected in 7 out of 58 (12%) diagnostic myeloma samples and in 2 out of 52 other hematological samples (1 MPD and 1 B-ALL). None of the MGUS or ‘*normal*’ bone marrow controls showed aberrant methylation of *PTEN*. Representative MSP results are shown in Figure 1A.

When methylation results were analyzed for potential correlations with MM clinical pathologic characteristics including age, B2 microglobulin, lactate dehydrogenase (LDH), serum creatinine, hemoglobin and calcium levels, type of protein, type of light chain, Salmon Durie tumor stage and cytogenetic abnormalities, no statistically significant correlations were found, except for a lower level of LDH (*P* = 0.01). Altogether, patients with aberrant *PTEN* status had a prevalence of M component IgG, Salmon Durie stage I, and lower LDH levels values at the time of sampling and were classified as intermediate/standard cytogenetic risk, except for a single high-risk patient (Table 1).

When PTEN mRNA levels were measured to verify whether *PTEN* gene hypermethylation could determine loss of PTEN expression, results showed that the expression levels in MM samples differed significantly from those of non-neoplastic tissues (mean value of -0.72 ± 14 versus 0.016 ± 0.30, respectively; *P* <0.001) and MGUS (-0.72 ± 1.4 versus 0.14 ± 0.32, respectively; *P* = 0.0013). No statistically significant correlation between loss of *PTEN* expression and clinical parameters were found except for the LDH (*P* = 0.001) and serum creatinine (*P* = 0.02) levels (data not shown).

Furthermore, comparison of PTEN expression levels in hypermethylated and unmethylated MM subgroups did not reach a statistically significant difference with mean values of 0.002 ± 0.23 and -0.03 ± 0.44, respectively (*P* = 0.85). Nevertheless, there was a tendency to lower levels in the hypermethylated subgroup (Figure 1C,D).

Survival analysis did not show a statistically significant difference between the hypermethylated and unmethylated subgroup (*P* = 0.53). Thus, *PTEN* epigenetic status did not have a prognostic impact on multiple myeloma patients. However, the median overall survival of patients with *PTEN* methylation was longer that those with an unmethylated promoter (median OS 91.8 versus 34.7 months).

## Discussion

*PTEN* is involved in cellular differentiation, reproduction and apoptosis, as well as cellular adhesion and mobility. The loss or downregulation of PTEN plays an important role in the multiple steps of tumorigenesis and progression of human malignancies. In hematological malignancies, *PTEN* promoter was found hypermethylated in 20% of acute lymphoblastic leukemia and chronic myeloid leukemia cases, and it is also involved in the mechanism determining imatinib resistance [17]. Our findings show that *PTEN* is hypermethylated in a small proportion of MM patients, indicating that epigenetic modification of *PTEN* also may have a role in MM gene inactivation mechanism besides mutations and gene deletions. This MM subgroup had a prevalence of M component IgG, Salmon Durie stage I, and intermediate/standard cytogenetic risk. Moreover*, PTEN* hypermethylation is linked with low LDH level values when compared with those of the unmethylated subgroup. We did not observe any major effect of *PTEN* hypermethylation on overall survival, even though MM subgroup patients with aberrant *PTEN* had a tendency to superior survival with respect to unmethylated patients according to their clinical stage and laboratory parameters. We have hypothesized that other factors, such as regimen treatments, may have a role. In agreement with other studies, we found abnormal expression of *PTEN* in myeloma primary samples, but in our cohort, low levels of *PTEN* expression were associated with low levels of LDH and serum creatinine, signs of a less aggressive disease. In addition, we found that *PTEN* deregulation is independent or weakly associated with its epigenetic status, suggesting a lack of functional consequence to *PTEN* methylation. There is evidence that in leukemia samples *PTEN* expression does not correlate with functional inactivation and other defects in which regulatory mechanisms may act.

In conclusion, *PTEN* hypermethylation is observed in MM samples, but it affects neither gene expression nor clinical outcome. Since *PTEN* is one of the potential targets along the PI3K/Akt pathway, the modulation of its expression is a potential strategy for treatment of MM [18-20], and further studies are needed to investigate the *PTEN* inactivation process in multiple myeloma.

## Abbreviations

AML: acute myeloid leukemia; B-ALL: B lymphoblastic leukemia; BM: bone marrow; CML: chronic myeloid leukemia; FISH: fluorescent *in situ* hybridization; LDH: lactate dehydrogenase; MPD: myeloproliferative disorders; MGUS: monoclonal gammopathy of undetermined significance; MM: multiple myeloma; MSP: methylation specific polymerase chain reaction; OS: overall survival.

## Competing interest

The authors declare that they have no competing interests.

## Authors’ contributions

GP participated in the design of the study, performed laboratory work, statistical analysis of data and wrote the manuscript; MM participated in the design of the study and contributed to writing the manuscript; ADP and AC participated in clinical data collection; FV, LP and RA participated in laboratory work and methylation experiments; AG participated in the design of the study and contributed to the final version of the manuscript; GCL was responsible for all aspects of the study. All authors read and approved the final version of the manuscript.
